# Large-scale phenotypic and genomic analysis of *Listeria monocytogenes* reveals diversity in the sensitivity to quaternary ammonium compounds but not to peracetic acid

**DOI:** 10.1128/aem.01829-24

**Published:** 2025-03-04

**Authors:** Mirena Ivanova, Martin Laage Kragh, Judit Szarvas, Elif Seyda Tosun, Natacha Friis Holmud, Alexander Gmeiner, Corinne Amar, Claudia Guldimann, TuAnh N. Huynh, Renáta Karpíšková, Carmen Rota, Diego Gomez, Eurydice Aboagye, Andrea Etter, Patrizia Centorame, Marina Torresi, Maria Elisabetta De Angelis, Francesco Pomilio, Anders Hauge Okholm, Yinghua Xiao, Sylvia Kleta, Stefanie Lüth, Ariane Pietzka, Jovana Kovacevic, Franco Pagotto, Kathrin Rychli, Irena Zdovc, Bojan Papić, Even Heir, Solveig Langsrud, Trond Møretrø, Phillip Brown, Sophia Kathariou, Roger Stephan, Taurai Tasara, Paw Dalgaard, Patrick Murigu Kamau Njage, Annette Fagerlund, Frank Aarestrup, Lisbeth Truelstrup Hansen, Pimlapas Leekitcharoenphon

**Affiliations:** 1 Research Group for Genomic Epidemiology, National Food Institute, Technical University of Denmark 114320 , Kgs Lyngby, Denmark; 2 Research Group for Food Microbiology and Hygiene, National Food Institute, Technical University of Denmark 114320 , Kgs Lyngby, Denmark; 3 Public Health England, National Infection Service 371011 , London, United Kingdom; 4 Chair for Food Safety and Analytics, Ludwig-Maximilians-University Munich, Munich, Germany; 5 University of Wisconsin-Madison 5228 , Madison, Wisconsin, USA; 6 Department of Public Health, Masaryk University, Medical Faculty, Brno, Czech Republic; 7 University of Zaragoza 16765 , Zaragoza, Spain; 8 University of Vermont 2092 , Burlington, Vermont, USA; 9 Istituto Zooprofilattico Sperimentale dell'Abruzzo e del Molise G Caporale “Giuseppe Caporale” 83371 , Teramo, Italy; 10 Arla Innovation Center, Arla Foods amba, Aarhus N, Denmark; 11 German Federal Institute for Risk Assessment (BfR), National Reference Laboratory for Listeria monocytogenes (NRL-Lm), Berlin, Germany; 12 Austrian Agency for Health and Food Safety (AGES), Institute of Medical Microbiology and Hygiene, National Reference Laboratory for Listeria monocytogenes 31329 , Graz, Austria; 13 Food Innovation Center, Oregon State University, Portland, Oregon, USA; 14 Listeriosis Reference Service, Food Directorate, Bureau of Microbial Hazards, Ottawa, Ontario, Canada; 15 Unit for Food Microbiology, Institute for Food Safety, Food Technology and Veterinary Public Health, University of Veterinary Medicine 572163 , Vienna, Austria; 16 Institute of Microbiology and Parasitology, Veterinary Faculty, University of Ljubljana 54767 , Ljubljana, Slovenia; 17 Nofima, The Norwegian Institute of Food, Fisheries and Aquaculture Research, Ås, Norway; 18 North Carolina State University 6798 , Raleigh, North Carolina, USA; 19 Institute for Food Safety and Hygiene, Vetsuisse Faculty, University of Zurich 600627 , Zurich, Switzerland; The Pennsylvania State University, University Park, Pennsylvania, USA

**Keywords:** *Listeria monocytogenes*, food industry, disinfectants, quaternary ammonium compounds, peracetic acid

## Abstract

*Listeria monocytogenes* presents a significant concern for the food industry due to its ability to persist in the food processing environment. One of the factors contributing to its persistence is decreased sensitivity to disinfectants. Our objective was to assess the diversity of *L. monocytogenes* sensitivity to food industry disinfectants by testing the response of 1,671 *L*. *monocytogenes* isolates to quaternary ammonium compounds (QACs) and 414 isolates to peracetic acid (PAA) using broth microdilution and growth curve analysis assays, respectively, and to categorize the isolates into sensitive and tolerant. A high phenotype-genotype concordance (95%) regarding tolerance to QACs was obtained by screening the genomes for the presence of QAC tolerance-associated genes *bcrABC*, *emrE*, *emrC,* and *qacH*. Based on this high concordance, we assessed the QAC genes’ dissemination among publicly available *L. monocytogenes* genomes (*n* = 39,196). Overall, QAC genes were found in 23% and 28% of the *L. monocytogenes* collection in this study and in the global data set, respectively. *bcrABC* and *qacH* were the most prevalent genes, with *bcrABC* being the most detected QAC gene in the USA, while *qacH* dominated in Europe. No significant differences (*P* > 0.05) in the PAA tolerance were detected among isolates belonging to different lineages, serogroups, clonal complexes, or isolation sources, highlighting limited variation in the *L. monocytogenes* sensitivity to this disinfectant. The present work represents the largest testing of *L. monocytogenes* sensitivity to important food industry disinfectants at the phenotypic and genomic level, revealing diversity in the tolerance to QACs while all isolates showed similar sensitivity to PAA.

**IMPORTANCE:**

Contamination of *Listeria monocytogenes* within food processing environments is of great concern to the food industry due to challenges in eradicating the isolates once they become established and persistent in the environment. Genetic markers associated with increased tolerance to certain disinfectants have been identified, which alongside other biotic and abiotic factors can favor the persistence of *L. monocytogenes* in the food production environment. By employing a comprehensive large-scale phenotypic testing and genomic analysis, this study significantly enhances the understanding of the *L. monocytogenes* tolerance to quaternary ammonium compounds (QACs) and the genetic determinants associated with the increased tolerance. We provide a global overview of the QAC genes prevalence among public *L. monocytogenes* sequences and their distribution among clonal complexes, isolation sources, and geographical locations. Additionally, our comprehensive screening of the peracetic acid (PAA) sensitivity shows that this disinfectant can be used in the food industry as the lack of variation in sensitivity indicates reliable effect and no apparent possibility for the emergence of tolerance.

## INTRODUCTION

*Listeria monocytogenes* is a major foodborne pathogen causing listeriosis, a deadly infectious disease that affects individuals with weakened immune systems, elderly, neonates, and is responsible for miscarriages in pregnant women (1). Unlike salmonellosis and campylobacteriosis, the two leading bacterial causes of foodborne illnesses, listeriosis has a low incidence but a high mortality rate (2). The major cause of human listeriosis is consumption of contaminated ready-to-eat (RTE) foods (3).

Food contamination with *L. monocytogenes* most often occurs in food production facilities where *L. monocytogenes* enters with raw materials, personnel, or equipment, and can establish and persist for decades in food processing environments (FPEs), or it can be regularly introduced into FPEs with incoming raw materials (4). Combinations of genetic (biotic) and environmental (abiotic) factors contribute to the successful survival and persistence of *L. monocytogenes* in FPEs. These factors include the ability to form biofilms, growth at low temperatures and in low-nutrient environments, increased tolerance to disinfectants and desiccation, poor sanitation practices, among others, which can lead to contamination with and survival of subtypes with increased persistence potential (5, 6).

To help control *L. monocytogenes* in FPEs, effective and robust cleaning and disinfection (C&D) programs must be established. Within C&D programs, three major disinfectant categories are widely applied, namely quaternary ammonium compounds (QACs), peroxygens, and halogen-releasing agents (7–9), and used in different combinations and alternations.

Among them, QACs are the most common non-oxidizing disinfectants, consisting of a cationic quaternary nitrogen and an alkyl chain of varying lengths. The cationic quaternary nitrogen interacts with head groups of the acidic phospholipids and the negatively charged structural proteins of the cytoplasmic membrane, leading to membrane breakage, leakage of cytoplasm, and cell lysis (10). It has been shown that the length of the alkyl chain affects the inhibitory ability of the QACs. The longer the alkyl chain, the stronger the antibacterial activity (11, 12).

Efflux pump genes, located either on plasmids (e.g., *bcrABC*, *emrC*) or in the chromosome (e.g., *qacH*, *emrE*, *fepA*, *sugE1*/*sugE2*), are the main genetic mechanisms described to increase QAC tolerance. The *bcrABC* cassette, consisting of a TetR family transcriptional regulator (BcrA) and two small multidrug resistance (SMR)-type efflux pumps BcrB and BcrC, is the most widespread QAC tolerance mechanism due to its location on plasmids with various genetic contexts in many *L. monocytogenes* clonal complexes (CCs) (13, 14). Another commonly detected QAC tolerance gene, *qacH*, belonging to the SMR protein family of efflux pumps, in the majority of the cases is chromosomally encoded, located on the Tn*6188* transposon (15). *qacH*-like genes located on plasmids have also been recently identified (16, 17). Other efflux pump genes, such as *mdrL*, *lde* (18, 19), or mutations in efflux pump repressor genes (*fepR*, *sugR*) and their promoters have also been reported to cause increased tolerance to QACs in adaptation experiments (20, 21).

Many studies have emphasized that the level of efflux pump-mediated tolerance to QACs or the increased tolerance that *L. monocytogenes* can develop in adaptation experiments are irrelevant under food industry conditions, as in-use QAC concentrations in FPEs (200–1,000 mg/L or ppm) are more than 100–200 times higher than the level of disinfectant tolerance that *L. monocytogenes* can develop. The relevance of the increased tolerance, however, either by carrying a QAC gene(s) and/or by developing a mutation(s) upon disinfectant exposure, is important for instances where QACs are inappropriately used or ineffectively removed from the FPEs. Such residual QAC concentrations may aid survival of *L. monocytogenes* subtypes carrying tolerance genes and increase their persistence potential and virulence (7, 22, 23).

In contrast to the QACs, few studies have linked specific genetic determinants with increased *L. monocytogenes* tolerance toward strong oxidizing disinfectants, such as peracetic acid (PAA) or sodium hypochlorite (9, 24, 25), probably due to their complex modes of action, including oxidation, di-hydroxylation of double bonds, and free-radical formation (26) with less possibility to develop tolerance. Kragh et al. (27) have recently examined the sensitivity of 240 *L*. *monocytogenes* isolates to PAA by the broth microdilution method and showed no variation in the minimum inhibitory concentration (MIC) values, determined to be 62 mg/L for all isolates. In contrast, another study using a bactericidal suspension test reported a wide variation in the log reductions (average 2 log CFU/mL, range 0–6 log CFU/mL) of the analyzed 588 *Listeria* spp. isolates from pre-harvest and post-harvest environments (28) after 30 s exposure to 80 mg/L PAA.

Considering the above-mentioned limitations, in this study we aimed to assess the diversity in *L. monocytogenes* sensitivity to two commonly applied food industry disinfectants, QACs and PAA, in a large and diverse collection of 1,671 isolates and to elucidate phenotype-genotype concordance. The broth microdilution method was employed to test sensitivity to two pure QAC substances, benzalkonium chloride (BC) and didecyl dimethyl ammonium chloride (DDAC), and to a commercial QAC-based disinfectant, while sensitivity to PAA was tested by the broth microdilution assay and growth curve analysis. For the QACs, the observed high phenotype-genotype concordance (95%), linking the carriage of one of the four QAC genes (*bcrABC*, *emrC*, *emrE,* or *qacH*), and an increased QAC tolerance, led to the final objective of the study, to determine the global distribution of these four genes in publicly available *L. monocytogenes* raw sequence data (*n* = 39,196) deposited in the European Nucleotide Archive (ENA) and their association with CCs, isolation sources, and geographic locations.

## RESULTS

### *L. monocytogenes* isolate collection

**Fig 1:**
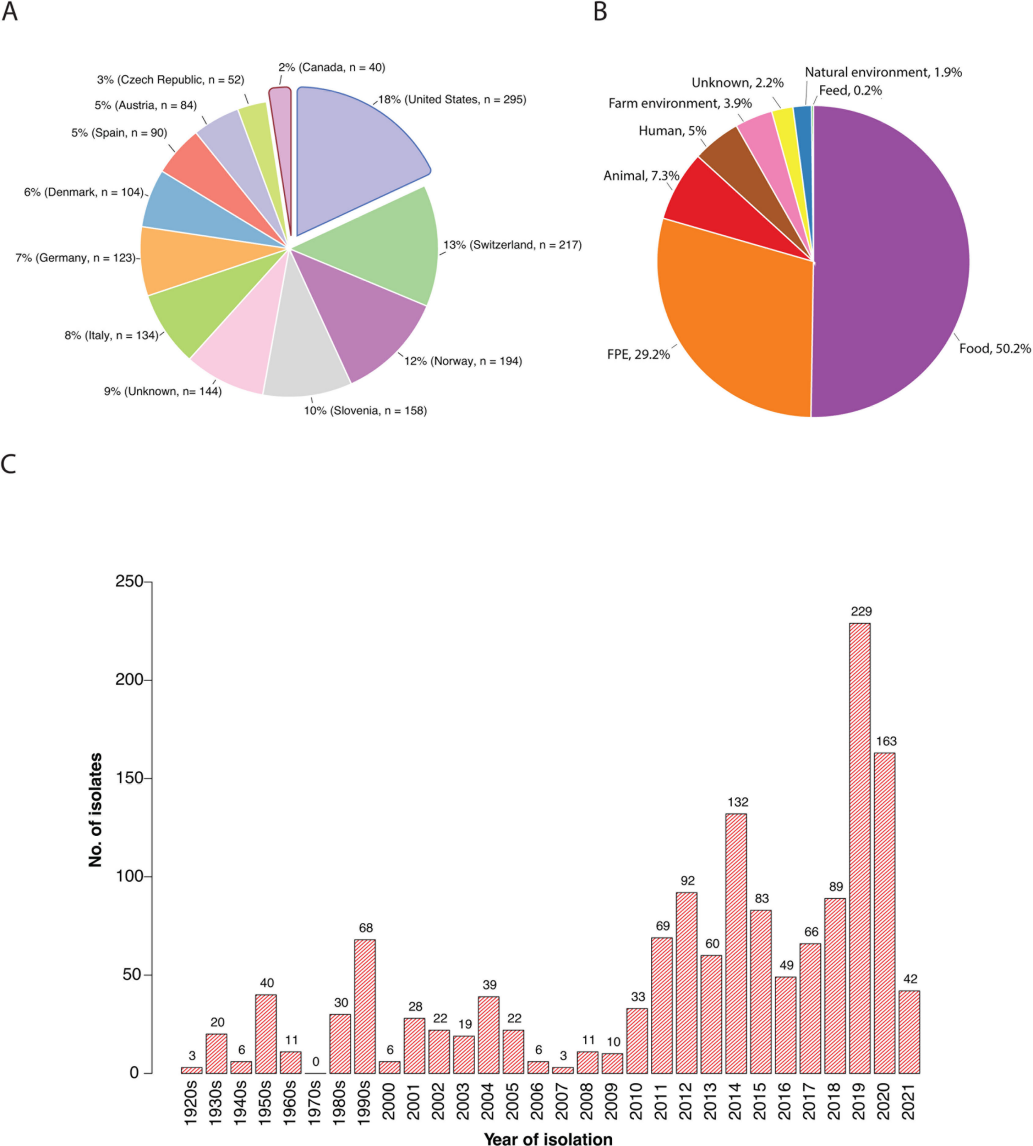
Distribution of the *L. monocytogenes* isolates by (**A**) country of isolation, (**B**) isolation source, and (**C**) year. In (**B**), countries with <1% isolates are not included in the graph (Romania [*n* = 16], UK [*n* = 10], Turkey [*n* = 3], Russia [*n* = 3], Belgium [*n* = 1], China [*n* = 1], Finland [*n* = 1], and France [*n* = 1]). In (**C**), 220 isolates with an unknown year of isolation are not shown in the graph.

For this study, we assembled a collection of 1,671 *L*. *monocytogenes* isolates from different geographic regions, isolation sources, and years to account for potential diversity in the *L. monocytogenes* disinfectant tolerance. The isolates were collected from Europe (*n* = 1,186; 71%), North America (the United States and Canada, *n* = 335; 20%), Russia (*n* = 2), Turkey (*n* = 3), China (*n* = 1) and belonged to seven isolation sources (Fig. 1A). The majority of isolates were isolated from food (*n* = 839; 50.2%) and FPEs (*n* = 488; 29.2%) (Fig. 1B). Isolates from diverse environments and countries were included as the stresses (environmental, antimicrobial, human, etc.) faced by *L. monocytogenes* vary, which could affect the disinfectant tolerance and adaptation of isolates from different niches. Additionally, the isolates were recovered within a time span of 98 years, from 1924 to 2021 (Fig. 1C).

**Fig 2:**
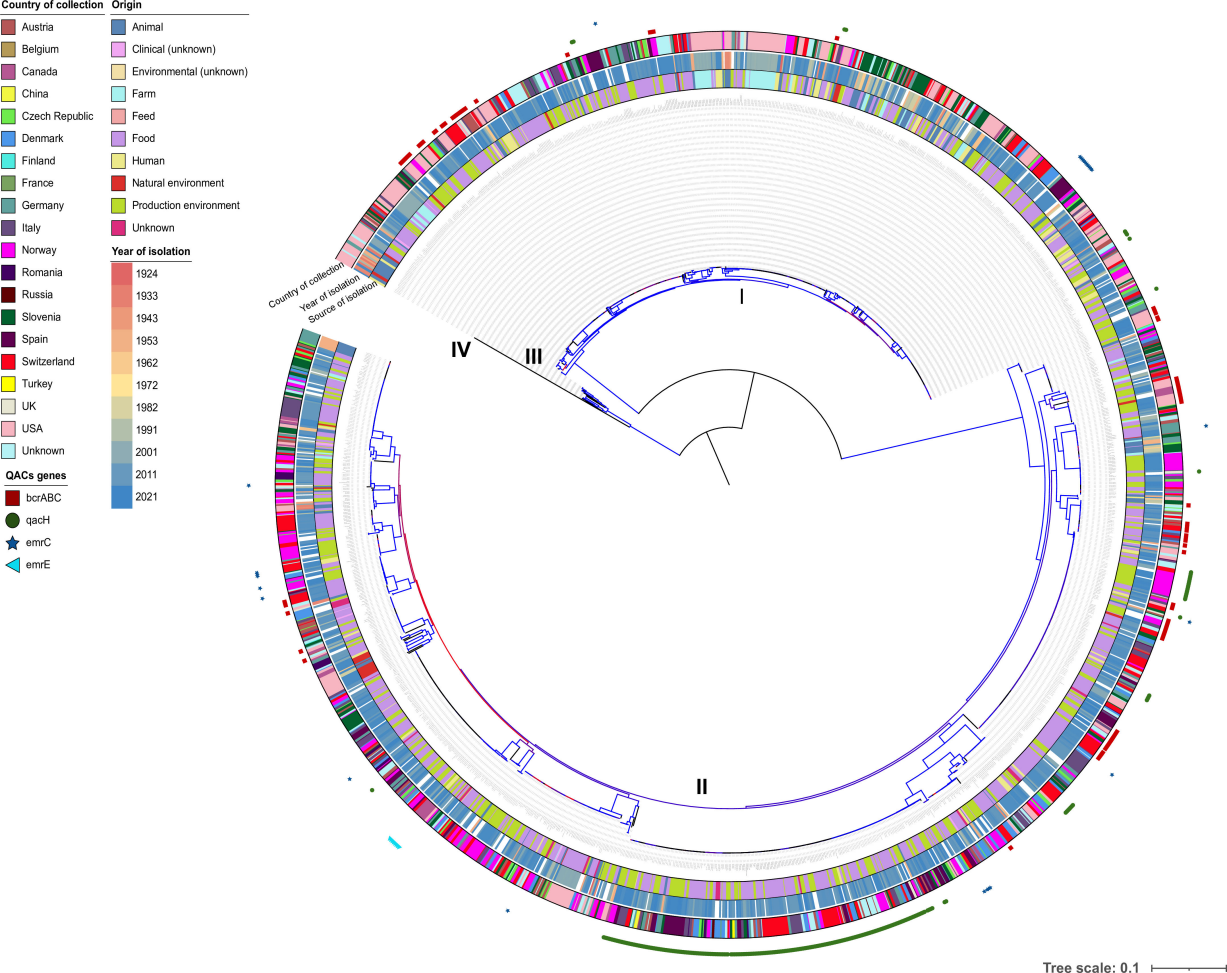
Mid-rooted maximum likelihood tree constructed from 2.19 Mb core-genome alignment using IQ-TREE with 1,000 ultrafast bootstraps and GTR+G nucleotide substitution model. The color of the branches represents bootstrap support values, from 50 (red) to 100 (blue). Roman numbers represent the *L. monocytogenes* phylogenetic lineages.

Single nucleotide polymorphism (SNP) analysis divided the 1,671 *L*. *monocytogenes* isolates into four evolutionary genetic lineages, i.e., lineage I (LI, *n* = 589; 35%), lineage II (LII, *n* = 1,064; 64%), lineage III (LIII, *n* = 17, <1%), and lineage IV (LIV, *n* = 1, <1%) (Fig. 2). Conventional multi-locus sequence typing (MLST) separated the isolates into 75 CCs and 20 singleton sequence types (STs). Among them, 11 CCs predominated, accounting for 67% (*n* = 1,117) of all isolates.

Distribution of the *L. monocytogenes* isolates by country of isolation, isolation source, and year is shown in Fig. 2.

### Sensitivity of 1,671 *L. monocytogenes* isolates toward quaternary ammonium compounds and phenotype-genotype concordance

**Fig 3:**
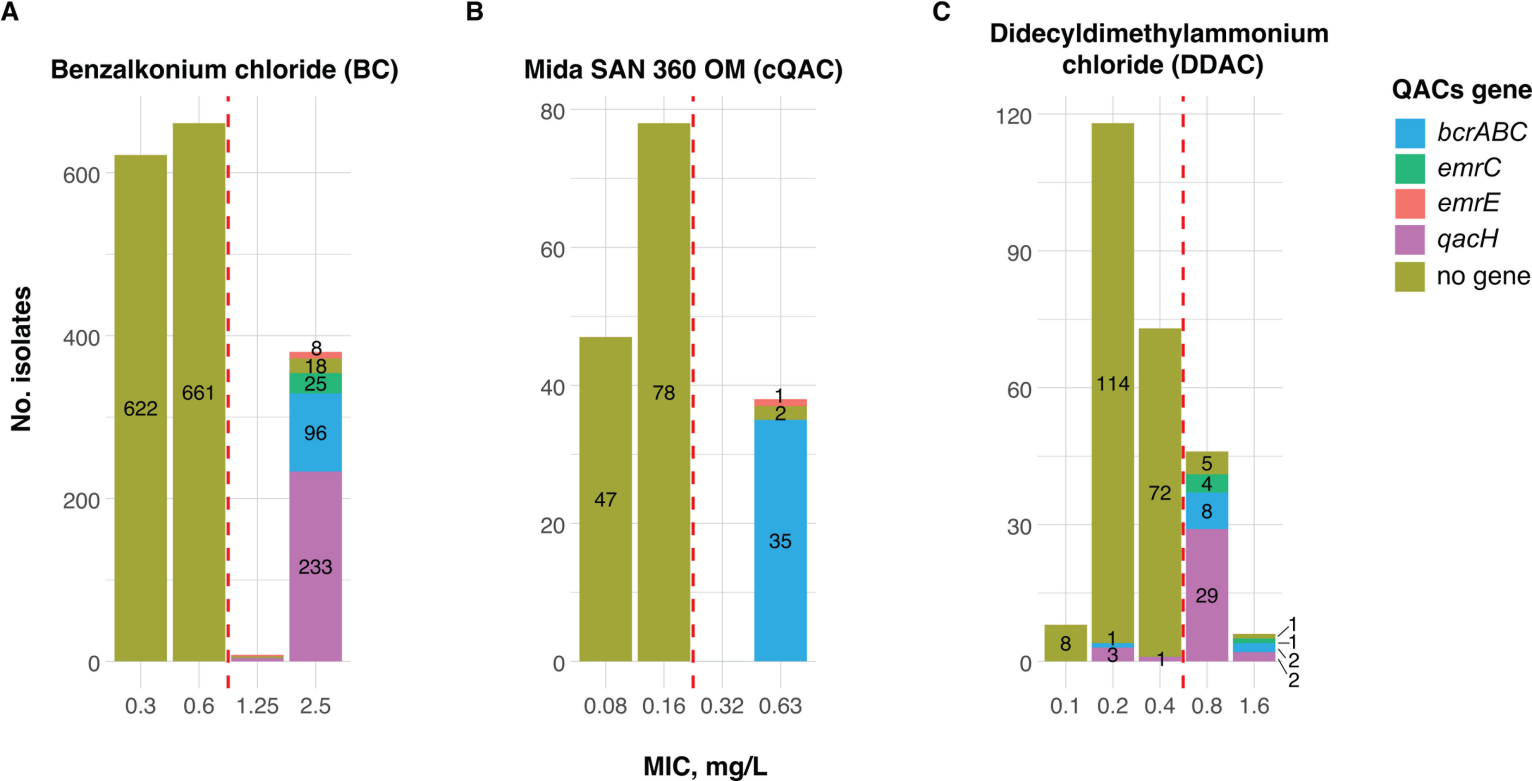
Distribution of the MIC values of (**A**) benzalkonium chloride, (**B**) Mida SAN 360 OM (cQAC), and (**C**) DDAC for the 1,671, 163, and 251 *L*. *monocytogenes* isolates tested, respectively. In (**A**), at MIC = 1.25 mg/L, the distribution of the isolates is as follows: *qacH* = 4, *emrE* = 2, and no gene = 2. Dashed lines represent the cut-off values established for this study and divide the isolates into sensitive and tolerant for each disinfectant.

All *L. monocytogenes* isolates (*n* = 1,671) were screened for BC tolerance, while the sensitivity to Mida San 360 OM (cQAC, *n* = 163) and DDAC (*n* = 251) was tested for selected isolates (Fig. 3; Table S1). Bimodal distributions of the MIC values were observed for both BC and cQAC based on the cut-offs for tolerance, which were established for this study. For BC, a cut-off of MIC ≥ 1.25 mg/L for tolerance was defined (Fig. 3A), where isolates with MICs < 1.25 mg/L were classified as BC sensitive (77%, *n* = 1,283) and isolates with MICs ≥ 1.25 mg/L as BC tolerant (23%, *n* = 388). Among the tolerant *L. monocytogenes* isolates (*n* = 388), 368 harbored one of the four screened QAC genes (*bcrABC*, *emrC*, *emrE,* or *qacH*), resulting in 95% phenotype-genotype concordance for tolerance to BC. The 20 BC tolerant isolates, which lacked any of the four QAC genes, were further examined for the presence of other efflux pump genes and mutations in their regulatory regions. The results of these analyses are detailed below.

Similarly to BC, a cut-off of MIC > 0.16 mg/L for tolerance to cQAC (Mida San 360 OM) divided the tested isolates into sensitive (MIC ≤ 0.16 mg/L, 77%, *n* = 125) and tolerant (MIC > 0.16 mg/L, 23%, *n* = 38) (Fig. 3B). Isolate Lm327 (CC9, ground beef, USA) with sensitive phenotype (MIC_cQAC_ = 0.16 mg/L) was tolerant to BC (MIC = 2.5 mg/L), but no QAC genes were detected, while all isolates with cQAC tolerant phenotypes harbored a QAC gene (100% concordance for tolerance to cQAC).

Bimodal MIC distribution was not observed for DDAC. Of the 199 isolates with MIC_DDAC_ ≤ 0.4 mg/L, 194 did not harbor any of the four QAC genes and were defined as DDAC sensitive. However, five isolates with MIC_DDAC_ ≤ 0.4 mg/L harbored a QAC gene*—bcrABC* in one isolate and *qacH* genes in four isolates (Table S1). In contrast, 49 of the isolates with MIC_DDAC_ > 0.4 mg/L (*n* = 52) harbored one of the four QAC genes and were defined as DDAC tolerant (94% phenotype-genotype concordance). Three isolates with tolerant phenotype (MIC_DDAC_ > 0.4 mg/L) did not harbor a QAC gene (Fig. 3C).

Furthermore, none of the isolates defined as tolerant to the three QAC disinfectants tested in this study carried more than one of the four screened QAC tolerance genes.

### Sensitivity of 414 *L. monocytogenes* isolates to peracetic acid

**Fig 4:**
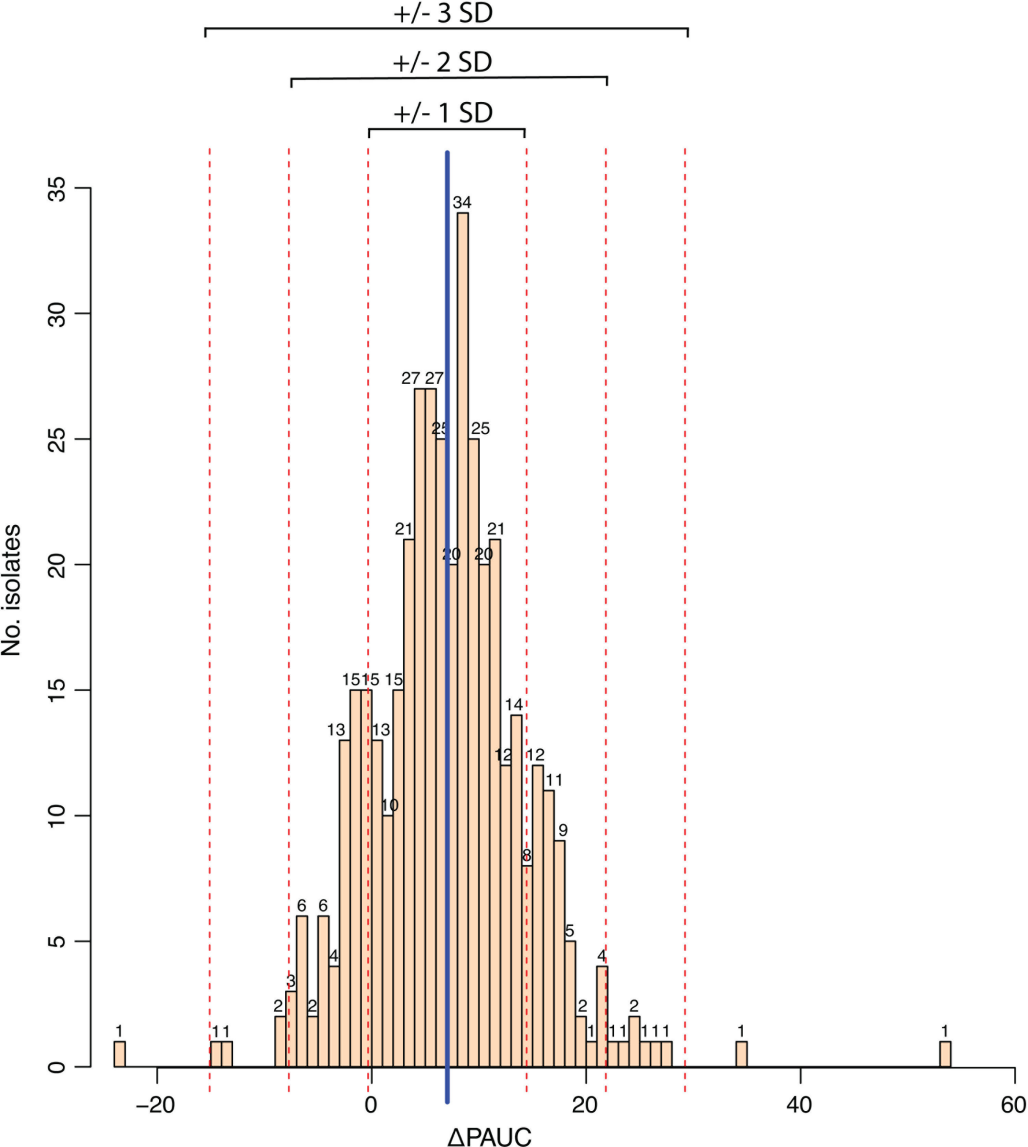
Overall distribution of the percentage change in area under the curve (ΔPAUC) for peracetic acid-induced effect on the growth of 414 *L*. *monocytogenes* isolates displaying a normal distribution with a mean ΔPAUC of 7.06% (±7.3 SD).

Initial testing of *L. monocytogenes* sensitivity to PAA using the broth microdilution method resulted in no variation in the MIC values (MIC = 63 mg/L) (data not shown). To further explore the seemingly absent diversity in the sensitivity to this disinfectant, a subset of 414 *L*. *monocytogenes* isolates, chosen to represent various isolation sources and countries of isolation, was tested using the more sensitive growth curve analysis method, i.e., growing *L. monocytogenes* with and without presence of PAA (31 mg/L, 0.5× MIC) and calculating percentage change in the area under the curve (ΔPAUC) between treated and untreated samples (Table S2). Results showed that ΔPAUCs for the 414 isolates were normally distributed with a mean percentage change of 7.06% (±SD 7.3%) (Fig. 4). The ΔPAUCs for 10 isolates were two SDs above the mean, indicating a more sensitive phenotype, while six isolates had ΔPAUCs with two SDs below the mean indicating an increased tolerance to PAA. Interestingly, 7 of the 10 isolates with the more sensitive phenotype were CC3, while the isolates with the tolerant phenotype belonged to various CCs (Table S2).

**Fig 5:**
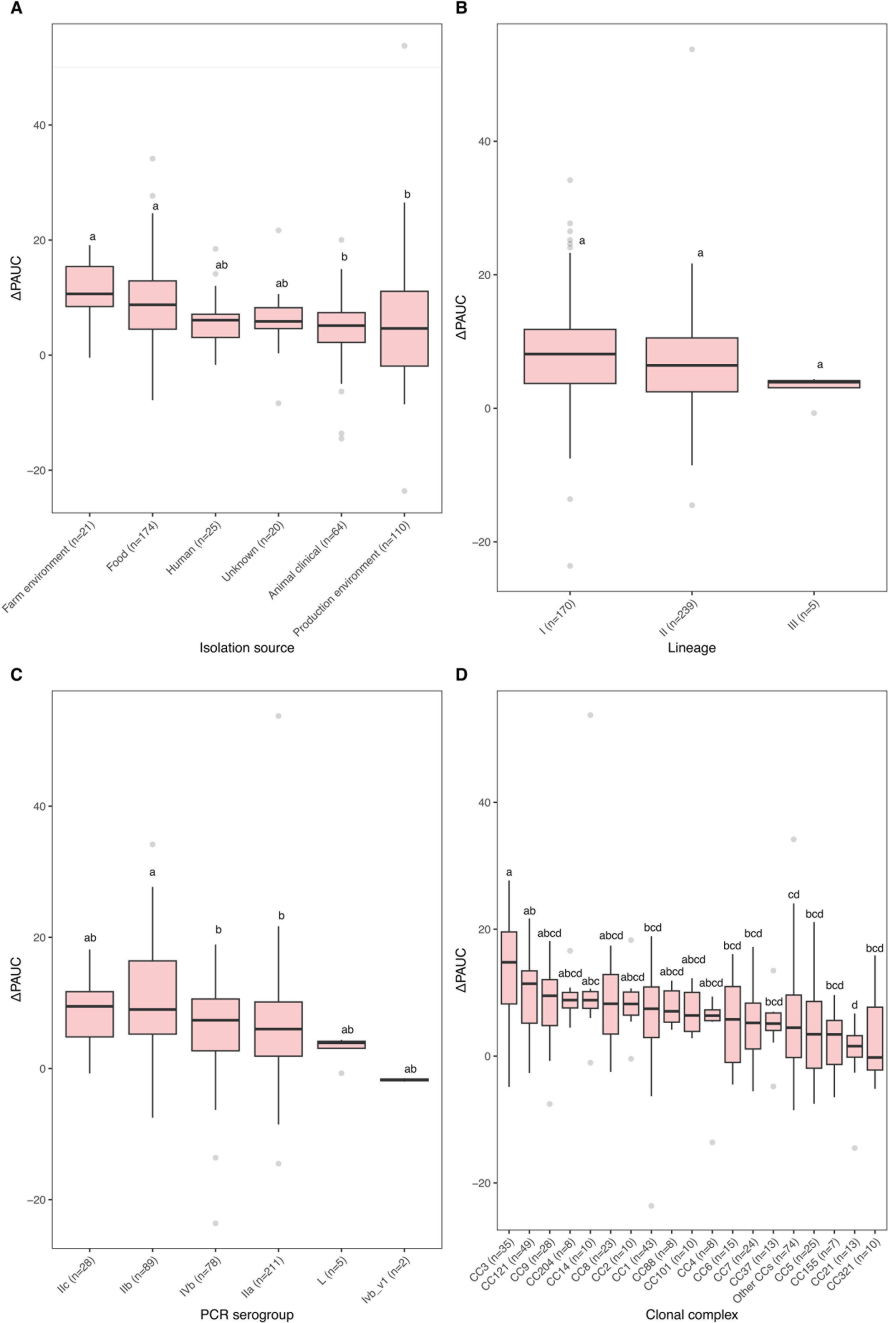
Peracetic acid effect on ΔPAUC comparisons between *L. monocytogenes* isolates grouped according to (**A**) isolation source, (**B**) phylogenetic lineage, (**C**) PCR serogroup, and (**D**) clonal complex. Groups not sharing a letter are significantly (*P* < 0.05) different. “Other CCs” category includes CCs with ≤5 isolates.

Further statistical analysis of the ΔPAUC values caused by the sub-lethal PAA stress revealed differences among isolates based on the isolation source, serotype, and CC. Regarding isolation source, farm environment and food isolates were significantly (*P* < 0.05) different from animal clinical and production environment isolates (Fig. 5A). In addition, there were no significant (*P* > 0.05) differences in the ΔPAUC between LI, LII, and LIII strains (Fig. 5B). PCR serogroup IIb displayed a significantly (*P* < 0.05) lower tolerance compared to IIa and IVb PCR serogroups (Fig. 5C). CC3 isolates were significantly (*P* < 0.05) different from CC1, CC6, CC7, CC37, other CCs, CC5, CC155, CC21 and CC321, while CC21 isolates were significantly (*P* < 0.05) different from CC14, CC121, and CC3 (Fig. 5D).

### Genomic characterization of the QAC-tolerant *L. monocytogenes* isolates

**Fig 6:**
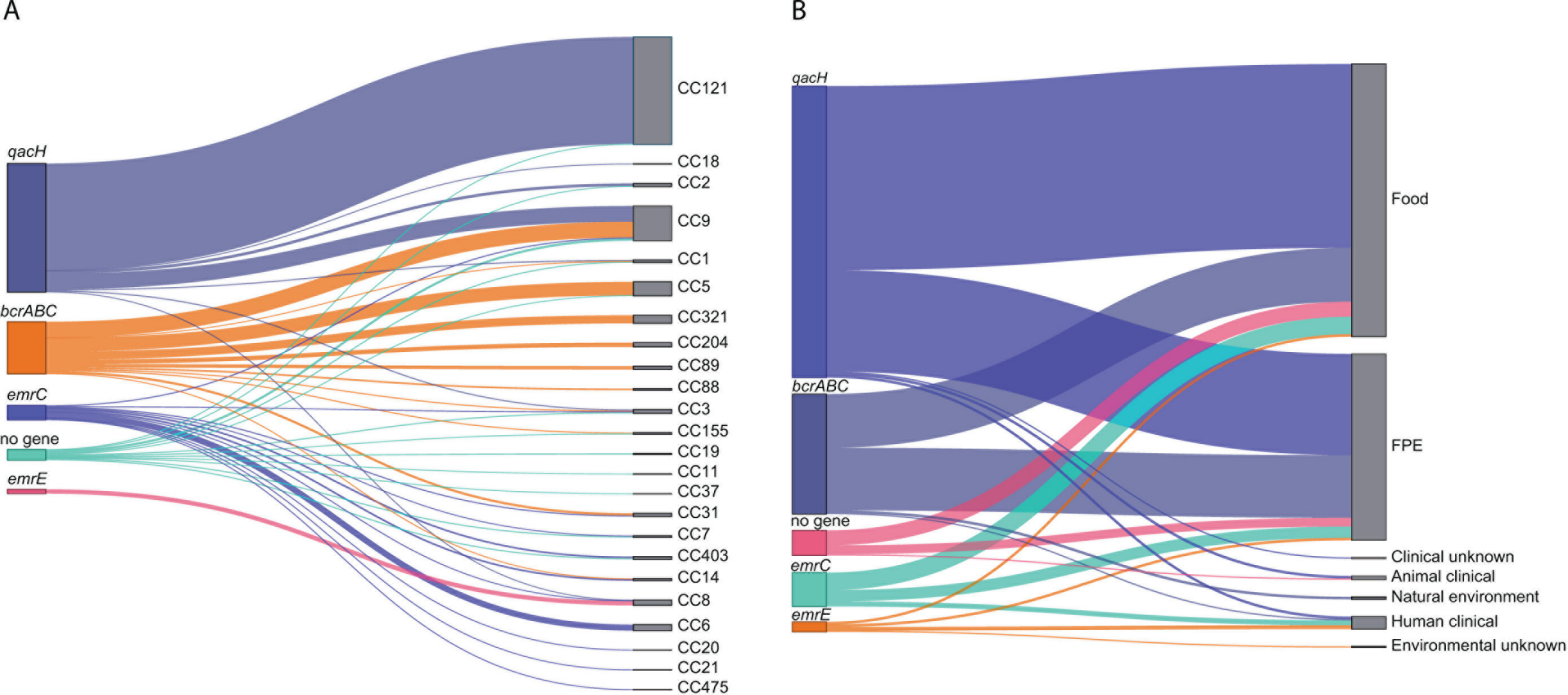
Distribution of the *bcrABC*, *emrC*, *emrE,* and *qacH* harboring *L. monocytogenes* isolates, including tolerant isolates with no known QAC gene according to (**A**) CC and (**B**) isolation source.

Of the four examined QAC tolerance genes, *qacH* was the most prevalent gene in our collection of isolates, present in 61% (*n* = 237) of the BC tolerant isolates, followed by *bcrABC* (25%, *n* = 96), *emrC* (7%, *n* = 27), and *emrE* (2%, *n* = 8). The majority of the *qacH*-harboring isolates belonged to CC121 (83%) and CC9 (12%), while the *bcrABC* genes were detected in 12 CCs, of which CC9 (30%), CC5 (26%), and CC321 (16%) dominated. Despite being present in only 27 isolates in our isolate collection, the *emrC* gene occurred in multiple CCs (*n* = 10), and among them, CC6 (41%), CC14 (11%), and CC403 (11%) dominated. The *emrE* gene was detected only in CC8 isolates. Overall, most of the isolates harboring QAC genes were LII isolates (85%), of which 60% belonged to CC121 and 20% to CC9, while no QAC genes were detected among LIII and LIV isolates (Fig. 6A). The QAC genes were mostly found in isolates from food (56%) and FPE (38%) sources, and only 3% were detected in isolates from clinical sources (Fig. 6B).

**Fig 7:**
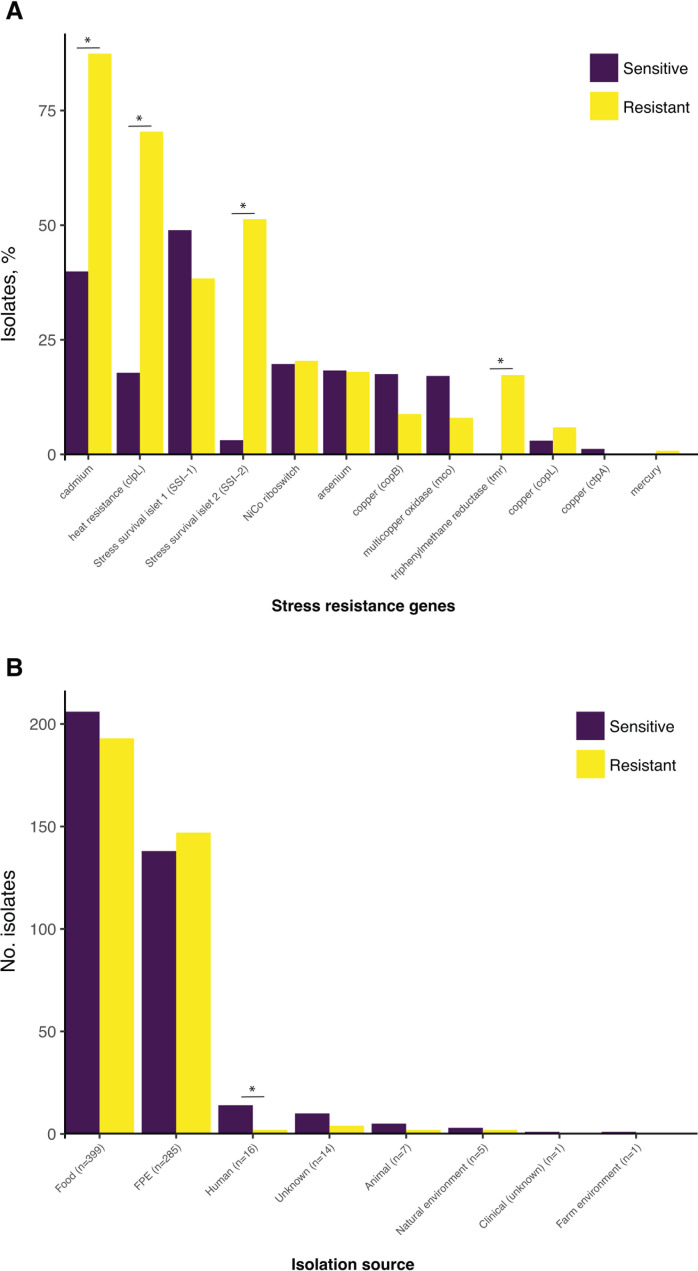
(A) Prevalence of stress resistance genes among the benzalkonium chloride (BC)-tolerant (*n* = 388) and sensitive (*n* = 1,283) isolates. (**B**) Plasmid-harboring BC-sensitive and -tolerant *L. monocytogenes* isolates divided by isolation source. Asterisks indicate significant differences determined with the Pearson’s chi-squared association test with *P* < 0.05. Details on the stress resistance gene screening are given in Table S5.

The *bcrABC* cassette was detected on contigs that carried plasmid-associated genes, which corresponded with its distribution among many CCs compared to *qacH* and *emrE*. The *emrC* gene was located on short plasmid contigs (4.4–4.7 kbps), comprising seven open reading frames. *emrE* as part of the LGI-1 was located on the chromosome in all isolates. The *qacH* gene was predominantly (97.5%, 231 of 237) located on contigs with no identified replicon gene and lengths greater than the largest identified *Listeria* spp. plasmid (>152 kbp, CP022021.1), assuming chromosomal origin. Blastn using a custom database of *L. monocytogenes* stress resistance genes against the contigs carrying replicon genes found *qacH* and *repA* genes co-located in six G4 plasmid group isolates. In these isolates, *qacH* had different genetic context, consisting of *tetR* and *qacH* with varying identities to the same genes on the chromosome, and a transcriptional regulator *mutR* (Fig. S1). *qacH* had the highest nucleotide diversity among the four QAC genes, and subsequently, seven *qacH* gene variants were observed in our isolate collection, of which variant 7 (*n* = 182) was exclusively present in CC121, and all but two variant 9 isolates belonged to CC9 (Fig. S2). In addition, there was a significantly (*P* < 0.05) higher prevalence of cadmium resistance genes, triphenylmethane reductase gene *tmr*, heat resistance gene *clpL,* and stress survival islet 2 gene (SSI-2) in BC tolerant as compared to BC sensitive isolates (Fig. 7A). Except for human clinical isolates, there was no significant difference in the plasmid content between QAC-tolerant and sensitive isolates (Fig. 7B).

### Genomic characterization of the 20 tolerant *L. monocytogenes* isolates that lacked any of the four QAC tolerance genes

The 20 QAC-tolerant isolates, in which neither *bcrABC*, *emrC*, *emrE,* or *qacH* was detected, were genetically diverse, belonging to 12 CCs (Fig. 6A). All of them originated from FPEs or RTE food except isolate L295, which was recovered from an animal clinical sample (Fig. 6B). An additional screening of these isolates against a database consisting of all reported efflux-pump genes involved in increased QAC tolerance (Table 1) confirmed their presence in all 20 isolates. Previous studies have emphasized the importance of mutations in *fepR* and *sugR*, transcriptional regulators of *fepA* and *sugE1*/*sugE2*, respectively, and their promoter regions for increased QAC tolerance in adaptation experiments (20–22). SNPs or indels in the *fepR* promoter region were identified in 8 of the 20 isolates compared to the EGDe *fepR* promoter reference sequence (Fig. S3). To determine if the observed SNPs were present only in QAC-tolerant isolates and potentially explain the tolerant phenotype, all QAC-sensitive isolates were screened for the presence of these SNPs, and the result showed that all SNPs and combinations of them were also detected in sensitive isolates (data not shown). Additionally, SNPs were identified in the *fepR* gene in 12 of the 20 isolates (Fig. S4). Of them, the SNPs were non-synonymous in five of the isolates, and a single nucleotide insertion was detected in one isolate, all those leading to a PMSC and potentially non-functional FepR. SNPs in the *sugR* promoter region were identified in 14 isolates compared to the *sugR* promoter sequence of *L. monocytogenes* EGD-e, four of which were either in the −10 or −35 promoter regions (Fig. S5). Isolate N20-2732 harbored three SNPs in its *sugR* promoter sequence, which were also present in four QAC-sensitive strains isolated from the same country (Switzerland). The rest of the SNPs in the *sugR* promoter sequee were ubiquitously present in QAC-sensitive isolates (data not shown). No PMSCs were detected in the *sugR* gene, but four amino acid substitutions were observed, none of which have previously been reported to enhance QAC tolerance (Fig. S6).

**TABLE 1:** Genes and mutations reported to confer tolerance to QACs in *L. monocytogenes*

Gene	Protein and location	Function	References
*bcrABC*	The BcrABC cassette, consisting of a TetR family transcriptional regulator (BcrA) and two SMR-type efflux pumps BcrB and BcrC, has been found on various plasmids in *Listeria*	Associated with increased tolerance specifically to QACs	29
*qacH*	The efflux pump QacH, part of the SMR family, is located on Tn*6188* on the chromosome; QacH-like efflux pumps located on plasmids have also been reported	Associated with increased tolerance to QACs and EtBr	15, 17
*emrE*	EmrE is an efflux pump located on *Listeria* genomic island 1 (LG1)	Associated with increased tolerance specifically to QACs	30
*emrC*	Efflux pump EmrC located on pLMST6	Associated with increased tolerance specifically to QACs	31
*fepR* ^*a*^	FepR is a local repressor of the fluoroquinolone efflux pump FepA	Mutations in *fepR* have been associated with BC adaptation	20, 32, 33, 34
*fepA*	FepA belongs to the multidrug and toxic compound extrusion (MATE) family	Removes QAC substances from the bacterial cytosol	35
*sugR* ^*a*^	The transcriptional regulator SugR belongs to the TetR family and regulates the expression of the efflux pumps SugE1 and SugE2	Mutations in *sugR* have been associated with CTAB adaptation	20, 35
*sugE1, sugE2*	SugE1 and SugE2 belong to the SMR-type efflux pumps	BC induced expression; *sugE1*/*sugE2* mutants show decreased MIC to QACs	20, 35
*dgkB*	Diacylglycerol kinase DgkB converts diacylglycerol to phosphatidic acid, used for synthesis of phospholipids	Mutations in *dgkB* lead to alterations in the cell membrane composition—possible adaptation mechanism in *L. monocytogenes* to QACs	20
*mepA, norM*	MepA and NorM belong to the MATE efflux pumps	Not QAC-specific; efflux of QACs, aminoglycosides, and fluoroquinolones	36, 37
*lde, mdrL*	Multidrug resistance *Listeria* (Lde) and *Listeria* drug efflux (MdrL) belong to the major facilitator superfamily efflux pumps	Not QAC-specific; Lde is associated with resistance to fluoroquinolones, acridine orange, and EtBr; MdrL is additionally involved in resistance to macrolides, cefotaxime, and heavy metals; *mdrL* is regulated by *ladR*	18, 19

^*a*^ Mutations in *fepR* and *sugR* promoter regions have been described to lead in overproduction of the efflux pumps FepA and SugE1/SugE2 and to increased tolerance to QACs in adaptation studies (20, 33).

### Occurrence of QAC tolerance genes among global *L. monocytogenes* isolates

**Fig 8:**
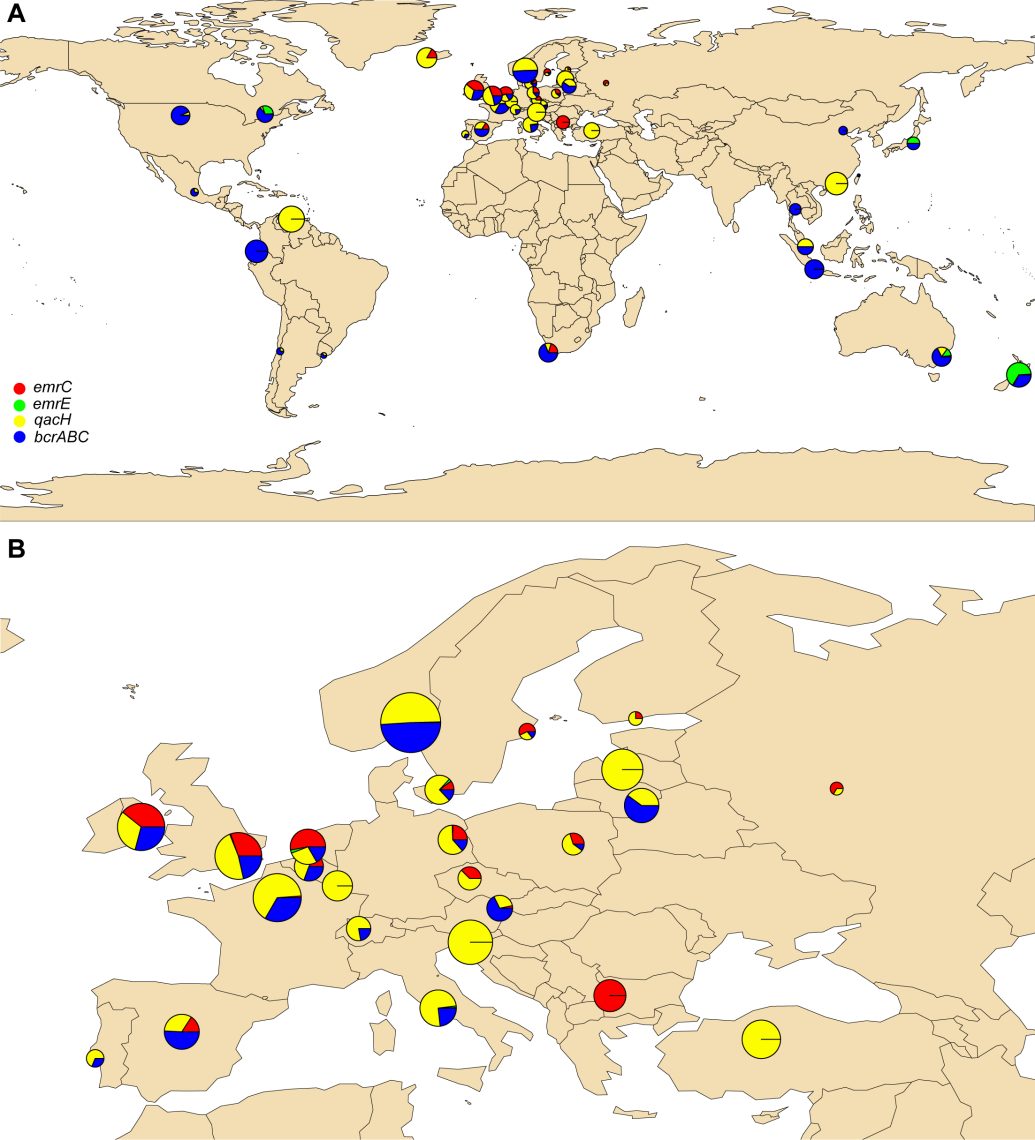
Distribution of *bcrABC*, *emrC*, *emrE,* and *qacH* genes (**A**) globally and (**B**) in Europe in *L. monocytogenes* sequencing runs deposited in ENA as of 29 April 2021. The pie charts indicate the proportion (rate) of the genes in each of the country in which at least one of the genes was present, and their size reflects the rate of each gene per 1,000 genomes. The pie sizes represent the number of QAC-tolerant *L. monocytogenes* isolates.

Due to the high phenotype-genotype concordance (95%) for the QAC category of disinfectants, we assessed the occurrence and distribution of *bcrABC*, *emrC, emrE,* and *qacH* among global *L. monocytogenes* isolates. We downloaded and screened raw sequence data of 39,196 *L*. *monocytogenes* isolates deposited in the ENA until April 2021 for presence of the four QAC genes (Fig. 8; Table S3). Overall, of the analyzed data with available country of isolation, 65% were deposited by the United States, followed by the United Kingdom (UK, 13.2%), and 12.5% by Europe (other than UK). In regard to the isolation sources, FPEs (42%) and food and feed (29.9%) categories prevailed among the isolates.

The occurrence of *bcrABC*, *emrC, emrE,* and *qacH* in the global *L. monocytogenes* raw sequence data were 28% (10,953 sequences contained QAC genes, and of them, 10,931 contained one QAC gene per genome). The *bcrABC* cassette was the most common genetic determinant, present in 72% of the QAC-positive isolates. *qacH* (19%) was the second most prevalent gene, followed by *emrC* (7%) and *emrE* (2%).

Interestingly, 22 isolates appeared to carry simultaneously two QAC genes. Their genomes were assembled, sub-typed by MLST, and re-screened for the presence of the four QAC genes. After assembly, seven out of the 22 assemblies carried only one QAC gene, and three had two alleles in an MLST gene, suggesting contamination of the raw reads. For the rest of the sequences (*n* = 12), the presence of two QAC genes cannot be reliably confirmed due to low coverage depth, except for isolate ERR2521912, where the presence of both *bcrABC* and *emrC* is possible (Table S4).

Difference in the dissemination of the QAC genes among countries/continents was also observed. The *bcrABC* cassette was globally detected; however, it was the dominating QAC gene in the United States. Of the 72% of the sequences that contained *bcrABC* in the global data set, 84% were present in *L. monocytogenes* sequences deposited by the United States. *qacH* and *emrC* were mainly detected in isolates recovered in Europe, and interestingly *emrE* was found in *L. monocytogenes* recovered in Australia/Oceania and Japan in addition to Canada (Fig. 8; Fig. S7 and S8).

The dissemination of the four QAC genes among CCs also varied. *qacH* was significantly associated almost entirely with CC121. The *emrC* and *emrE* genes were mainly detected in CC6 and CC8, respectively, while the *bcrABC* cassette was distributed among 25 CCs, having the highest occurrence in CC5 followed by CC321, CC155, CC9, and CC7; however, *bcrABC* was not detected in CC121 (Fig. S9).

In regard to isolation sources, all four QAC genes were significantly (*P* < 0.05) associated with isolates from FPE and food/feed sources as opposed to the clinical or natural environment. In contrast, *emrC* was significantly (*P* < 0.05) associated with isolates from both food/feed and clinical origins (Fig. S10).

## DISCUSSION

While disinfectant tolerance in *L. monocytogenes* has been widely researched (25, 38–41), genomic and phenotypic studies that employ diverse and large data sets to determine disinfectant tolerance cut-offs and genetic determinants responsible for increased disinfectant tolerance have not yet been undertaken. In this study, we collected 1,671 *L*. *monocytogenes* isolates with as diverse as possible origins and geographic locations in Europe and North America to assess the diversity in *L. monocytogenes* sensitivity to two of the most common disinfectants in the food industry, QACs and PAA.

An important bottleneck in comparing phenotypic data obtained by different studies is the lack of standard sensitivity testing methods. Hence, large variations in the obtained data are observed even if the same method is utilized, meaning that variations do not necessarily reflect differences in the tolerance level but are rather due to differences in the assay design. The same assay optimized by different laboratories could vary in, e.g., biocide and inoculum concentrations, medium types and brands, incubation temperatures (e.g., 37°C or lower food industry-relevant temperatures) and times, etc. (23, 39, 42–44). The importance of our large-scale disinfectant sensitivity study is the consistency and reliability of the obtained phenotypic data using the same assay and conditions, which allowed us to establish cut-offs for QAC tolerance for this study, divide the *L. monocytogenes* isolates into sensitive and tolerant, and elucidate the genetic basis for the observed tolerance. The high QAC phenotype-genotype concordance in this study (95%) (i.e., tolerant phenotype and presence of either *qacH*, *bcrABC*, *emrC,* or *emrE*) allows whole-genome sequencing (WGS)-based prediction of the increased tolerance without the need to conduct laborious wet-lab phenotypic testing. Such high concordance (100%) was also reported in a US study assessing sensitivity of 359 *L*. *monocytogenes* produce isolates to QACs, where the isolates with increased MICs (≥25 mg/L) harbored *bcrABC*, *qacH,* or *emrC* gene and were regarded as tolerant, while those with MIC ≤ 10 mg/L did not carry any of these genes and were sensitive (13).

Identification of tolerant *L. monocytogenes* isolates that lack known QAC genes has also been observed in other studies (32, 38, 45). While some of the detected amino acid substitutions in efflux pumps and/or SNPs in their regulatory regions and combinations of them (Table 1) might contribute to the increased QAC tolerance in the 20 isolates with unknown mechanisms of tolerance in this study, mutation G178T in *fepR* leading to amino acid substitution E60* has previously been linked to increased QAC tolerance (33). Moreover, the presence of other efflux pump genes associated with QAC tolerance (e.g., *fepA*, *sugE1*/*sugE2*, *mdrL*, *lde*) in both tolerant and sensitive isolates in this study indicates that differential expression regulation might play an important role in the tolerance mediated by these genes. Hereditary tolerance, as suggested by He et al. (13) due to previous exposure to QACs could be another possible explanation for the increased tolerance in the 20 isolates. In their study, adaptation experiments showed that QAC tolerance in sensitive *L. monocytogenes* isolates can increase to MIC levels of *bcrABC-*carrying tolerant isolates and retain its level throughout repeated subculturing without the presence of BC. However, no genetic modifications were reported to explain the increased tolerance. Modifications in the cell wall (45) or novel yet undescribed mechanism(s) could also be involved in the increased tolerance in these 20 isolates. Genome-wide association studies (GWAS), which have the power to link patterns in genotypes to the respective phenotypes, could potentially be applied to the data set in this study. For instance, previous GWAS performed on 197 *L*. *monocytogenes* isolates identified a prophage region located on a mobile genetic element strongly associated with BC tolerance (25). Similarly, but using a different approach, Karlsmose et al. (46) reported an increase in phage-associated genes following BC treatment of mixed *L. monocytogenes* communities. The latter, however, could also be caused by competitive interactions between the strains in the mixed community due to, e.g., limited nutrient conditions.

Genes conferring resistance to environmental stresses, including exposure to QACs, are often co-harbored on plasmids and can support *L. monocytogenes* adaptation and persistence in various environments (25, 47). The *bcrABC* gene, carried on various types of plasmids in *Listeria*, is often co-located with other stress resistance genes, e.g., heavy metals (*cadA1*, *cadA2*, *cadC1*, *cadC2*, *copB*, *mer* operon), NiCo riboswitch-*gbuC-npr*, heat resistance *clpL*, *mco*, etc. (48), which can provide *L. monocytogenes* with additional fitness advantages when such selective pressures are present in the environment (16). This is in line with our results, where the cadmium resistance genes, the heat resistance gene *clpL,* and the triphenylmethane reductase gene (*tmr*) were strongly associated with QAC tolerant phenotype. In addition, the chromosomally-encoded stress survival islet 2 (SSI-2), significantly associated with QAC tolerance in this study, has previously been found in *qacH*-carrying *L. monocytogenes* CC121 isolates and reported to enhance their survival under alkaline and oxidative stress conditions in FPEs (49). On the other hand, the lack of significant difference in the plasmid content between QAC-tolerant and sensitive isolates recovered from FPEs and food is supported by Fagerlund et al. (17), who observed no significant differences in the plasmid content between the persistent and non-persistent isolates in their study.

While two previous *in silico* studies have performed similar large-scale screening of QAC tolerance genes, they either focused on *L. monocytogenes* genomes from the United States (13, 50), from FPEs and food (50) or assessed the distribution of *bcrABC*, *emrC,* and *qacH* genes (13). Our study also explored the association of *emrE* with increased QAC tolerance in *L. monocytogenes* and detected its presence in isolates from Australia/Oceania and Japan in addition to its previous detection in Canadian isolates only (30). Thus, our global survey provides additional knowledge on the dissemination of all four QAC tolerance genes in *L. monocytogenes* isolates from isolation sources and geographic locations available in ENA, including geographic areas with scarce or absent disinfectant tolerance reports.

When surveying the geographic prevalence of specific QAC genes in *L. monocytogenes* in public databases, it should be noted that the deposited sequencing data in ENA are extensively biased toward Europe and North America. It is remarkable that despite the *bcrABC* being located on plasmids with various structures, several prevalence studies showed its negligible detection in Europe (51, 52). In contrast, North American surveys reported scarce prevalence of *qacH* (13, 53). As previously discussed, one possible hypothesis for the difference in the dissemination of the QAC genes among geographic locations is the circulating or adapted *L. monocytogenes* CCs in these areas (50). In the US, *qacH* has rarely been found in *L. monocytogenes* isolates probably due to the lower prevalence of CC121, while the higher prevalence of *bcrABC* is likely due to the wide dissemination of CCs associated with this gene cassette, e.g., CC5, CC321, CC155, CC7, CC9, and CC199 (14). The latter CCs are less reported in Europe, hence the lower finding of *bcrABC*. CC121 has been the most prevalent CC in Europe according to several large-scale studies (17, 45, 54) and since *qacH* is predominately linked to CC121, these studies report higher detection of *qacH* compared to the other QAC genes.

Other possible factors for the clear geographical separation of *L. monocytogenes* CCs (and the QAC genes) could be practices in the use of disinfectants throughout the years or even more complex factors such as the environment, farming practices, food production systems, and genotypes, resulting in *L. monocytogenes* clones that are more adapted to survive and persist in a certain geographic area or environmental niche (17, 55).

For other geographic locations (e.g., South Africa, Asia, South America, and Mexico), associations between QAC genes-harboring isolates and their respective metadata are difficult to establish due to the small number of *L. monocytogenes* sequences deposited and the lack of other reports for comparison. Nevertheless, the new observation of *emrE* in *L. monocytogenes* from Japan, New Zealand, and Australia, previously detected in Canada only (30), could be explained by the geographic approximation and trading patterns between North America and Oceania/Australia and Japan as contamination of raw materials with specific *L. monocytogenes* clones in close geographic areas could affect the dissemination of the QAC genes in those areas.

While our global *in silico* QAC gene screening resulted in the tentative finding of two QAC tolerance genes in 12 of the *L. monocytogenes* isolates, which was in line with previous reports (50, 56), only the sequence of ERR2521912 was of reliable quality to make this deduction. Fagerlund et al. (17) found no more than one QAC gene among the tolerant *L. monocytogenes* isolates in their in-house generated sequence data of 769 isolates, which concurred with observations for tolerant isolates in the present study of 1,671 isolates. An *in vitro* study, in which a *qacH*-carrying *L. monocytogenes* CC2 isolate was successfully transformed with an *emrC* gene, showed dual carriage is possible and even resulted in a small increase in the QAC tolerance (43). However, in nature, it is unknown if *L. monocytogenes* acquire any advantage by harboring more than one QAC tolerance gene. To note, *bcrABC* has never been found in CC121, which is almost entirely associated with *qacH*, potentially due to mutual exclusivity. Moreover, the *emrC* gene is located on a small high-copy plasmid, and its presence with another QAC gene could present a metabolic burden on the cell. Taken together, it appears that further research is needed to elucidate possible natural concurrent carriage of multiple QAC genes in *L. monocytogenes*.

Regarding the distribution of the ΔPAUC_PAA_ values of only a few *L. monocytogenes* isolates in our study into more tolerant (2%, 10/414) and more sensitive (1%, 6/414) groups based on the distance from the average ΔPAUC_PAA_ value, previous studies have published contrasting results. Wiedmann et al. (28) reported a wide variation in the log CFU/mL reduction for 588 *Listeria* spp. produce isolates after exposure to higher in-use PAA concentrations (80 mg/L) for a short time (30 s). However, no genomic data were available to support this phenotypic variation. Additionally, the undertaken approach in their study to test *L. monocytogenes* PAA sensitivity is fundamentally different from the broth microdilution assay in the study of Kragh et al. (27) and the growth curve analysis in this study, where either no or limited variation in PAA tolerance was observed. In terms of underlying mechanisms, it has been shown that a short-term exposure of *Escherichia coli* to biocides (including PAA) upregulates chaperones, which is a common cell response to various environmental stress conditions, while long-term exposure to PAA involved upregulation of specific genes for this biocide, e.g., biofilm formation genes (57). Thus, the growth curve assay would be a useful approach to study possible mechanisms of tolerance toward PAA in *L. monocytogenes* as it is expected that higher sub-lethal PAA concentrations would lead to an increased ΔPAUC diversity. Nonetheless, PAA is a disinfectant with high oxidizing power and multiple modes of action, and as such, it is unlikely to achieve increased tolerance or adaptation or to detect specific genes involved in its tolerance. Kastbjerg and Gram (58) for instance exposed *L. monocytogenes* EGDe to gradually increasing concentrations of a commercial disinfectant containing PAA and hydrogen peroxide for several hundred generations without any increase in the tolerance level. Similarly, no increase in the MIC and minimum bactericidal concentration values to PAA was observed in 124 persistent *L. monocytogenes* isolates recovered from a 4-year period in a factory using PAA as a disinfectant (41).

In conclusion, the occurrences of QAC tolerance genes in this study’s *L. monocytogenes* collection and in the global data set were 23% and 28%, respectively. High QAC phenotype-genotype concordance (95%) was observed (i.e., a presence of *bcrABC*, *emrC*, *emrE,* or *qacH* gene and a tolerant phenotype). Therefore, the presence of these four genes in *L. monocytogenes* can be used to predict the tolerance to low concentrations of QACs without performing phenotypic tests. The QAC tolerant phenotype in the remaining 5% of the *L. monocytogenes* could be explained by a previous QAC exposure (hereditary tolerance), differential expression regulation of efflux pump genes, or another yet undescribed mechanism of tolerance. Geographic differences in the global distribution of the QAC tolerance genes were observed and depended on the distribution of CCs in the given geographic area/environment. In contrast to the QACs, separation of the isolates into PAA tolerant and sensitive phenotypes was less clear due to limited variation in the normally distributed ΔPAUC_PAA_ values using the growth curve analysis assay under the sub-MIC PAA concentration used. Despite the seemingly limited PAA tolerance variation among *L. monocytogenes* isolates, GWAS could be a possible approach to explore genomic features associated with subtle differences in the PAA sensitivity for some of the *L. monocytogenes* isolates.

## MATERIALS AND METHODS

### *Listeria monocytogenes* isolates

A total of 1,671 *L*. *monocytogenes* isolates recovered from food (*n* = 839; 50%), FPE (*n* = 488; 29%), animal (*n* = 122; 7%), human (*n* = 83; 5%), farm environment (*n* = 66; 4%), unknown sources (*n* = 37; 2.2%), natural environment (*n* = 32; 1.9%), and feed (*n* = 4; 0.2%) were collected for this study (Fig. 1B). Most of the isolates (*n* = 1,336; 80%) originated from Europe (Switzerland, 13%; Norway, 12%; Slovenia, 10%; Italy, 8%; Germany, 7%; Denmark, 6%; Spain, 5%; Austria, 5%, and Czech Republic, 3%), while 18% of them were isolated in the United States and 2% in Canada (Fig. 1A). Additionally, the isolates were recovered within a time span of 98 years, from 1924 to 2021 (Fig. 1C).

### DNA extraction and whole genome sequencing

*L. monocytogenes* isolates that have not been previously whole genome sequenced (*n* = 1,244) were sequenced as part of this study. The isolates were grown on trypticase soy agar (TSA) at 37°C, overnight. A single colony per strain was transferred to 1.8 mL trypticase soy broth (TSB) and grown at 37°C overnight. Genomic DNA was extracted by the DNeasy Blood and Tissue Kit (Qiagen, Denmark) following the manufacturer’s recommendations except that the DNA was eluted in 10 mM Tris-HCl (pH = 8.5) (BioNordika, Denmark). The DNA concentration was measured using the Quant-iT dsDNA high sensitivity kit (Invitrogen, Denmark) by VICTOR X2 Multilabel Microplate Reader (Spectralab Scientific Inc.). Sequencing libraries were constructed using the Nextera XT Library Prep Kit (Illumina, San Diego, CA, USA), normalized and denatured for loading in a NextSeq 500/550 Mid Output v2.5 Kit (300 cycles) (Illumina), and pair-end sequenced on a NextSeq 500 platform (Illumina). The remaining 427 *L*. *monocytogenes* isolates were sequenced in previous works (Table S1).

### Assembly, species identification, *in silico* sub-typing, and core-genome phylogeny

The raw sequencing data of the 1,671 isolates were processed with the FoodQCpipeline v1.6 (https://bitbucket.org/genomicepidemiology/foodqcpipeline/src/master/), which uses bbduk2 from bbtools (Bushnell B. sourceforge.net/projects/bbmap/) for trimming and SPAdes (59) for genome assembly. Quality control of the sequence reads was performed before and after trimming by FastQC v0.11.5 (60). Quality of the assemblies was assessed by Quast v4.5 (61) and thresholds for number of contigs (≤300 contigs) and genome size (3 ± 0.5 Mb) were established. *In silico* species identification was performed using KmerFinder v2.0 (62). Assemblies were submitted to the BIGSdb-*L. monocytogenes* Pasteur MLST database (https://bigsdb.pasteur.fr/listeria/) for sub-typing (Table S1). Assemblies were further annotated with Prokka v1.14.6 referencing the genus *Listeria* and the species *monocytogenes* (63). Core-genome alignment was generated by Roary v3.13.0 (64) using the .gff files from Prokka as input, with 95% blastp identity threshold and paralog splitting (-s) disabled to prevent presumed paralogous genes from being split into different gene groups. The core-gene alignment was trimmed with trimAI (65) and option-gappyout to decide optimal thresholds based on the gap percentage count over the whole alignment. A maximum-likelihood phylogenetic tree was inferred using IQ-TREE v2.1.3 (66) with the GTR+G nucleotide substitution model and 1000 bootstrap replicates (--ufboot 1000) and mid-point rooted. The tree was visualized and annotated in iTOL (67).

### Determination of the *Listeria monocytogenes* sensitivity to QACs

MIC of *L. monocytogenes* to two pure biocide substances, BC (500 g/L, Thermo Fisher, Kandel, Germany), DDAC (500 g/L, Sigma-Aldrich, Denmark), and a commercial disinfectant—Mida SAN 360 OM (10%–15% 2-methoxymethylethoxy propanol, 3%–5% didecyl dimethyl ammonium chloride, <3% 1,2-ethanediol; Christeyns, Denmark) were tested by an in-house optimized broth microdilution assay according to Wiegand et al. (44) and Kragh et al. (27). The *L. monocytogenes* isolates were streaked from −80°C on trypticase soy agar plates (TSA; 40 g/L) and incubated at 37°C overnight. Three single colonies per isolate were transferred to 0.1× trypticase soy broth (TSB; 3 g/L) and cultured at 15°C for 48 h under stationary conditions until the optical density at 620 nm (OD_620_) reached ~0.1, (corresponding to 10^8^ CFU/mL). The final inoculum concentration was 10^5^ CFU/mL per well. The range of the disinfectant concentrations was selected according to previously reported MIC values (25) using twofold dilutions. A positive control consisting of inoculated 0.1× TSB and a negative control consisting of sterile 0.1× TSB were included in each 96-well plate. The plates were sealed with adhesive film (ThermoFisher, Denmark) and incubated at 15°C for 48 h. The MIC, defined as the lowest biocide concentration at which the *L. monocytogenes* growth was inhibited, was determined by measuring the *L. monocytogenes* OD_620_ by a Multiscan FC Microplate Reader (Thermo Scientific, Denmark). The threshold for growth was set at OD_620_ ≥0.08, which is 60%–65% of OD_620_ of the positive control and 200% of the negative control. The experiment was performed in two independent biological replicates with three technical replicates each. The result was considered valid if two out of the three technical replicates had identical MIC values. One twofold MIC variation between biological replicates was considered acceptable, and the higher MIC value was reported as the final result, unless the twofold dilution difference was at the cut-off for tolerance (e.g., MIC ≥ 1.25 mg/L). In this case, the test was repeated a third time.

### Determination of the *Listeria monocytogenes* sensitivity to PAA

Sensitivity of *L. monocytogenes* isolates to PAA (Sigma-Aldrich, Denmark) was initially tested by an in-house optimized broth microdilution assay as described in the previous section for QACs (except that the PAA concentration ranged 4–2,000 mg/L) and further by the more sensitive growth curve analysis as described by Wambui et al. (68). For the growth curve analysis assay, *L. monocytogenes* was grown in the same conditions described for the broth microdilution assays, diluted to 10^6^ CFU/mL and 50 µL used for inoculating 250 µL 0.1× TSB supplemented with 31 mg/L of PAA (treatment) or without PAA (control) in a 100-well honeycomb plate (Labsystems, Helsinki, Finland). Fresh PAA stocks were prepared prior to each experiment. PAA concentration of 31 mg/L was selected for the growth curve analysis as this was previously determined to be the sub-MIC of *L. monocytogenes* by the broth microdilution method (MIC = 63 mg/L) both in this study and by Kragh et al. (27). For each *L. monocytogenes* isolate, two technical replicates for the treatment and two for the control were included and incubated in the Bioscreen C plate reader (Labsystems, Finland) placed in an incubator with a programmed temperature of 15°C ± 0.1°C until stationary phase was achieved. The OD_540_ was measured every 30 min after shaking the culture. The growth curve parameters of the controls and treatments were calculated using the “opm” R package (69). Percentage change in the area under the curve (ΔPAUC) was calculated based on the difference in the AUC of the treated (31 mg/L, 0.5× MIC) and AUC of the control (no PAA) for each analyzed isolate.

### Gene screening for disinfectant tolerance, stress resistance, and plasmid replicon genes

All assemblies were initially screened for the presence of *bcrABC*, *emrC*, *emrE,* and *qacH* using minimum identification (--minid) and minimum coverage (--mincov) thresholds of 90% in ABRicate v1.0.1 (Seemann T, Abricate, Github https://github.com/tseemann/abricate). *L. monocytogenes* isolates that lacked *bcrABC*, *emrC*, *emrE,* or *qacH* were further screened for other QAC genes (Table 1) using ABRicate with minimum identification and coverage thresholds as indicated above. Furthermore, mutations in regulatory genes *fepR* and *sugR* and their promoter regions were identified by extracting their sequences using getfasta in bedtools v2.30.0 (70) and aligning them to the respective sequences of *L. monocytogenes* EGD-e as reference using MAFFT v1.5.0 in Geneious Prime 2023.0.4. The 1,671 *L*. *monocytogenes* isolates were additionally screened for stress resistance genes (Table S5) and plasmid replicon genes using ABRicate with minimum identification and coverage thresholds described above.

### Gene screening for disinfectant tolerance genes in global *L. monocytogenes* isolates

To study the global occurrence of the genes associated with increased tolerance to QACs (*emrC*, *emrE*, *bcrABC,* and *qacH*) in *L. monocytogenes*, publicly available sequencing data deposited in ENA as of 26 November 2018 were screened using COBS (COmpact Bit-sliced Signature index) v0.1.2. COBS consists of 661,405 assembled and indexed bacterial genomes, of which 26,006 were annotated as *L. monocytogenes* (71). COBS was run with default settings (97% nucleotide identity) for *emrE* (NC_013766.2:c1850670-1850347), *emrC* (MT912503.1:2384–2770), and *bcrABC* (JX023284.1) genes. Hits were manually examined, and only genes with ≥90% nucleotide coverage were considered present. Unlike *emrC*, *emrE,* and *bcrABC* genes, which sequences are conserved (>99% identity), the nucleotide diversity of *qacH* led us to reduce the nucleotide similarity to 90% to retrieve all *qacH* gene variants (Fig. S3). The STs of the *L. monocytogenes* isolates positive for QAC tolerance genes were obtained from the metadata associated with COBS.

Furthermore, pair-end raw sequencing runs of *L. monocytogenes* deposited in ENA between 27 November 2018 and 29 April 2021 were downloaded and screened for QAC tolerance genes using k-mer alignment with minimum template identity and coverage of 90% for all four QAC genes (72). Database consisting of the four QAC genes was indexed using k-mer = 16. The STs of the *L. monocytogenes* isolates harboring QAC genes were determined by stringmlst v0.6.3 (73) with k-mer = 35 using the *L. monocytogenes* MLST database and converted to CCs using Pasteur’s BIGSdb-Lm MLST database (https://bigsdb.pasteur.fr/). Only sequences determined as actually *L. monocytogenes* by stringmlst and which contained all seven MLST genes and one allele per gene were used for further data analysis.

### Statistical analysis

The heterogeneity in proportion of CCs, geographical locations, and isolation sources that were positive within and between genes was estimated using a random-effects model proposed by and implemented in the meta v4.4.1 R package (74, 75) to produce forest plots. Statistical heterogeneity within and between groups was estimated using the Cochran chi-square test and the Cochrane I^2^ index. The Pearson’s chi-squared association test was performed using R to determine statistically significant (*P* < 0.05) association between presence or absence of plasmids and stress survival genes in the phenotypes defined as tolerant or sensitive to BC. The ΔPAUC values of the isolates grouped by isolation source, phylogenetic lineage, serotype, and CC were compared using one-way analysis of variance in R with the Tukey post hoc test at a significance level of *P* < 0.05. All graphs were created using ggplot2 v3.5.1 R package (76), except the world map graph which was produced using rworldmap v1.3-6 R package (77) and the Sankey diagram using networkD3 v0.4 R package (78).

## Data Availability

The raw sequencing data have been deposited in the European Nucleotide Archive repository under the project accession number PRJEB56155. Overview of the metadata for the 1,671 isolates used in this study is presented in Table S1, and quality metrics (e.g., number of contigs, N50, etc.) are provided in Table QC.

## SUPPLEMENTAL MATERIAL

Supplemental figures aem.01829-24-s0001.docx Figures S1 to S10.

Supplemental tables aem.01829-24-s0002.xlsx Tables S1 to S5.

ASM does not own the copyrights to Supplemental Material that may be linked to, or accessed through, an article. The authors have granted ASM a non-exclusive, world-wide license to publish the Supplemental Material files. Please contact the corresponding author directly for reuse.
