# Erratum for Ivanova et al., “Large-scale phenotypic and genomic analysis of *Listeria monocytogenes* reveals diversity in the sensitivity to quaternary ammonium compounds but not to peracetic acid”

**DOI:** 10.1128/aem.00956-25

**Published:** 2025-06-03

**Authors:** Mirena Ivanova, Martin Laage Kragh, Judit Szarvas, Elif Seyda Tosun, Natacha Friis Holmud, Alexander Gmeiner, Corinne Amar, Claudia Guldimann, TuAnh N. Huynh, Renáta Karpíšková, Carmen Rota, Diego Gomez, Eurydice Aboagye, Andrea Etter, Patrizia Centorame, Marina Torresi, Maria Elisabetta De Angelis, Francesco Pomilio, Anders Hauge Okholm, Yinghua Xiao, Sylvia Kleta, Stefanie Lüth, Ariane Pietzka, Jovana Kovacevic, Franco Pagotto, Kathrin Rychli, Irena Zdovc, Bojan Papić, Even Heir, Solveig Langsrud, Trond Møretrø, Phillip Brown, Sophia Kathariou, Roger Stephan, Taurai Tasara, Paw Dalgaard, Patrick Murigu Kamau Njage, Annette Fagerlund, Frank Aarestrup, Lisbeth Truelstrup Hansen, Pimlapas Leekitcharoenphon

## ERRATUM

Volume 91, no. 4, e01829-24, 2025, https://doi.org/10.1128/aem.01829-24. Page 1: The affiliation number for Annette Fagerlund should be number 17 (Nofima, The Norwegian Institute of Food, Fisheries and Aquaculture Research, Ås, Norway).

