# Supplementary material for: Large-scale phenotypic and genomic analysis of Listeria monocytogenes reveals diversity in the sensitivity to quaternary ammonium compounds but not to peracetic acid
Source: Appl Environ Microbiol. 2025 Mar 4;91(4):e01829-24. doi: 10.1128/aem.01829-24 (PMC12016499; doi:10.1128/aem.01829-24)
Supplement: Supplemental figures — Figures S1 to S10. [file aem.01829-24-s0001.docx]

**Figure S1** Genetic organisation of the *qacH* gene located on the chromosome and plasmids. The chromosome-located *qacH* is part of the previously described Tn*6188* (15), while plasmid-located *qacH* has different genetic environment, consisting of *tetR* (72% nucleotide identity to *tetR* on the chromosome), *mutR* transcriptional regulator and genes encoding hypothetical proteins. The nucleotide identity between plasmid- and chromosome-located *qacH* was 92%.

**Figure S2** Multiple sequence alignment of the *qacH* variants detected among the *L. monocytogenes* isolates in this study. Additionally, two more *qacH* variants were detected within our collection (named 4.1 and 10 according to their phylogenetic placement). The result of the screening of the *qacH* gene variants is given in Table S1.

**Figure S3** Multiple sequence alignment of the *fepR* promoter region in 8 of the 20 *L. monocytogenes* isolates with unknown mechanisms of tolerance, carrying SNPs compared to EGD-e (AL591824).

**Figure S4** Multiple sequence alignment of the *fepR* gene in 12 of the 20 *L. monocytogenes* isolates with SNPs compared to the *fepR* gene of *L. monocytogenes* EGDe used as reference (GenBank accession number AL591824).

**Figure S5** Multiple sequence alignment of the *sugR* promoter region in 14 of the 20 *L. monocytogenes* isolates with unknown mechanisms of tolerance, carrying SNPs compared to *L. monocytogenes* EGD-e (GenBank accession number AL591824).

**Figure S6** Multiple sequence alignment of the *sugR* gene in 13 of the 20 *L. monocytogenes* isolates with SNPs compared to the *sugR* gene of *L. monocytogenes* EGD-e (GenBank accession number AL591824).

**Figure S7** Forest plot showing the association between QAC tolerance genes and country of isolation in the global *L. monocytogenes* dataset. “Unknown” indicates isolates which metadata lacked country of isolation/origin in ENA.

**Figure S8** Forest plot showing the association between QAC tolerance genes and continent in the global *L. monocytogenes* dataset. “Unknown” indicates isolates which metadata lacked country of isolation/origin in ENA.

**Figure S9** Forest plot showing the association between QAC tolerance genes and CC in the global *L. monocytogenes* dataset. “Unknown CC” indicates isolates with combination of alleles, which at the time of the analysis could not be assigned to a CC.

**Figure S10** Forest plot showing the association between QAC tolerance genes and source of isolation in the global *L. monocytogenes* dataset. “Unknown source” indicates isolates which metadata lacked isolation source in ENA.
